# Evaluating Potential Impacts of Climate‐Related Natural Disasters on Subsequent Prostate Cancer Mortality

**DOI:** 10.1002/cam4.71618

**Published:** 2026-02-11

**Authors:** Alexander P. Cole, Zhiyu Qian, Yu‐Jen Chen, Edoardo Beatrici, Rohit Acharya, Danesha Daniels, Prokar Dasgupta, Adam S. Kibel, Stuart R. Lipsitz, Quoc‐Dien Trinh, Hari S. Iyer

**Affiliations:** ^1^ Department of Urology Brigham and Women's Hospital, Harvard Medical School Boston Massachusetts USA; ^2^ Center for Surgery and Public Health Brigham and Women's Hospital, Harvard Medical School Boston Massachusetts USA; ^3^ Department of Urology Humanitas Research Hospital Milan Italy; ^4^ Common Good Labs Pittsburgh Pennsylvania USA; ^5^ Kings Health Partners London England; ^6^ Section of Cancer Epidemiology and Health Outcomes, Rutgers Cancer Institute New Brunswick New Jersey USA

**Keywords:** climate change, health services research, natural disaster, prostate cancer

## Abstract

**Background:**

Climate‐related disruptions to the health system may impact cancer outcomes. This may be particularly true for prostate cancer, which greatly contributes to cancer burden while existing on a risk spectrum, leading some men to delay treatment even in advanced cases.

**Methods:**

The study included men diagnosed with metastatic prostate cancer from 2010 to 2020 within SEER‐supported counties that experienced a climate‐related natural disaster from 2012 to 2018. A smaller subgroup of “major” disasters was classified based on individual assistance from FEMA. Year of natural disaster was considered the index date, with 2‐year pre‐ and post‐disaster periods compared. Age‐standardized incidence‐based metastatic prostate cancer mortality (IBM) rates were extracted from SEER and adjusted for demographics. Counties were then compared 147 SEER counties without any climate‐related natural disaster.

**Results:**

There were 222 counties across 11 states experiencing a single disaster within the study period, covering an estimated 27,787,120 people. Compared to the index year, prostate cancer IBM was 15% higher (RR: 1.15, 95% CI 1.02–1.30) 1‐year post‐disaster and 28% higher (RR: 1.28, 95% CI 1.11–1.49) 2 years post‐disaster. Associations were stronger among counties (*n* = 50) experiencing a “major” disaster (RR: 1.21, 95% CI: 1.05–1.40) and 35% (RR: 1.35, 95% CI: 1.17–1.55) at 1 and 2 years. In non‐exposed counties, this pattern was absent.

**Conclusions:**

We report a significant, dose‐dependent change in mortality from metastatic prostate cancer following a climate‐related natural disaster. The reasons are speculative but may include delayed diagnosis, care fragmentation, and interruptions for treatments for advanced disease including chemotherapy and radio‐hormonal therapy.

## Graphical abstract

Counties included in SEER registry and with corresponding data on FEMA‐designated natural disasters.

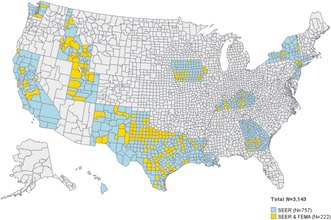

**Abbreviations**
FEMA: Federal Emergency Management Agency
IBM: incidence based mortality
PSA: prostate‐specific antigen
RR: rate ratio
SEER: surveillance, epidemiology, and end results

## 1 Introduction

Climate change is designated as the foremost threat to human health by both the United Nations (UN) and the World Health Organization (WHO) [1, 2]. Multiple indirect pathways involving extreme weather events and healthcare disruption may impact cancer pathogenesis, treatment, and outcomes [3, 4, 5]. Among cancers, prostate cancer is the most common solid organ cancer and second leading cancer‐related cause of death in men in the United States, leading to over 34,000 deaths and nearly 300,000 cases annually [6]. In the context of climate change, prostate cancer warrants special concern due to its long latency, high prevalence, and pathways involving access to care, potential environmental influences, and possible links to diet and adiposity that are influenced by climate‐related weather events [6, 7, 8, 9, 10].

Climate change may directly influence prostate cancer burden through changing diet and environmental exposures, though evidence is limited. Men living in regions with lower environmental quality, characterized by higher pollution levels and fewer natural resources, may have a higher risk of aggressive prostate cancer, though evidence is mixed [11, 12, 13, 14, 15, 16, 17, 18].

Hurricanes and extreme storms may flood industrial sites; this may lead to leaching of industrial chemicals into drinking water and agricultural areas [19]. For example, Hurricane Harvey exposed over 500 industrial sites in Texas to effects of flooding in 2017 [20]. Many of these industrial chemicals, including endocrine‐disrupting chemicals, have been linked to genitourinary cancers [21, 22, 23, 24, 25, 26, 27, 28, 29]. Changes in greenspace may also impact cancer risk: Observational epidemiologic studies in the United States and Canada comparing men living in neighborhoods with high versus low green space exposure have lower levels of inflammation, cardiometabolic stress, and risk of lethal prostate cancer in urban settings [13, 15, 30, 31, 32]. These inverse associations have been observed in other countries [33, 34, 35].

In addition to factors influencing prostate cancer development, climate change may produce more frequent extreme weather events, including hurricanes, floods, droughts, and landslides [36]. While direct medical impacts (e.g., heat stroke, dehydration, drowning, and traumatic injury) are the most obvious, extreme weather events can inundate low‐lying industrial areas and polluted sites with water, which may increase risk of chemical contamination in ground water and agricultural areas [21, 22, 23]. Extreme weather events can also interrupt care delivery. Studies have reported increases in mortality from cardiovascular disease and dementia following extreme weather events [37, 38]. For example, shortage of IV fluids after Hurricane Maria affected care even thousands of miles from the site of the hurricane [39, 40]. In late 2024, there were national IV fluid shortages caused by damage to manufacturing facilities by Hurricane Helene (North Carolina) and then Hurricane Milton (Florida). These shortages resulted in cancellation and delays of some surgical procedures [41, 42]. A recent study showed that individuals who initiated lung cancer radiation therapy within 30 days of a hurricane experienced roughly 30% higher mortality compared to those unaffected by the storm [43].

Prostate cancer is an ideal disease to study the impacts of climate‐related extreme weather events on cancer care. Even men with metastatic disease at diagnosis are increasingly living many years with modern systemic therapy and combined approaches that incorporate local therapy for low volume metastatic disease [44]. The disease has high prevalence and has other features that introduce climate vulnerabilities. These include the potential excellent prognosis with high quality care and the frequent intensive longitudinal assessments and treatments. These features have been shown to make prostate cancer survival highly sensitive to alterations in healthcare delivery and access to care (and therefore may plausibly be impacted by climate disasters) [45, 46, 47, 48]. We sought to compare population‐based trends in prostate cancer mortality before and after climate‐related natural disasters. We hypothesized that, in counties which experienced climate‐related natural disasters, there would be higher prostate cancer mortality 2 years following major natural disasters, with stronger associations among those with more severe natural disasters.

## 2 Methods

### 2.1 Data Sources

In compliance with the Strengthening the Reporting of Observational Studies in Epidemiology (STROBE) Guidelines, our research utilized annual cancer data from the Surveillance, Epidemiology, and End Results (SEER) registry from 2010 to 2020, capturing 47.9% of the US population. Annual mortality and incidence data from 22 geographic areas are reported by facilities, and clinical information is reviewed by registry officials for data quality and accuracy. Deaths are updated through linkages with the National Death Index, state and hospital records and other sources.

To identify climate‐related natural disasters, we used the publicly available Disaster Declarations Summaries provided by the Federal Emergency Management Agency (FEMA). This comprehensive dataset records every disaster that has been federally recognized and reported to FEMA since 1953. The dataset is updated in real time (every 20 min) and provides specific details such as the date, duration, and location of the event, the nature of the disaster (whether it be a major disaster or an emergency), the official title given to the event, and specific declared assistance programs (e.g., individuals and households, public assistance, hazard mitigation) [49].

### 2.2 Study Population and Design

Our goal in this study was to examine whether experiencing a weather‐related natural disaster was associated with higher county‐level prostate cancer mortality. Because counties in the United States vary dramatically with respect to sociodemographic factors, access to health care, and geographic factors, adequately controlling for confounding through measured variables alone is challenging. We therefore chose to compare prostate cancer mortality before and after the natural disaster using a county‐level self‐controlled design, a form of interrupted time‐series analysis [50]. This approach controls for measured and unmeasured time‐invariant confounding by factors such as racial composition, healthcare resources, and socioeconomic factors that are unlikely to change over the study period. Furthermore, because counties that experience natural disasters may differ dramatically from those that do not in terms of various unmeasured confounders, we felt that this self‐controlled design would provide a better causal inference compared to comparisons with unaffected counties which may differ significantly in their characteristics. A secondary analysis was also performed using incidence‐based mortality rates in the subset of SEER counties that did not experience any climate‐related natural disasters with complete covariate information (control counties). Time trends in these counties were compared to affected counties.

We included counties that experienced at least one natural disaster and included data from SEER for at least 2 years prior to and after the event. The 2‐year period prior to the event (pre‐disaster) served as a counterfactual baseline mortality rate for comparison with the post‐disaster period; the 2‐year period after the event was chosen as the minimal plausible time where we may be able to see an effect after the disaster. The male population residing in US counties that experienced a climate‐related natural disaster from 2012 to 2018 was included to allow follow‐up in the post‐disaster period through 2020.

### 2.3 Outcomes

Age‐adjusted incidence‐based mortality rates for metastatic prostate cancer were abstracted from each county and each year from 2010 to 2020. Incidence‐based mortality (IBM) rates are calculated by dividing the deaths (among incident cases) each year by the population at risk in that year. Because deaths are linked to cancer incidence data, IBM provides a more accurate estimate of the cause of death and allows for allocating the cause of death by stage and histologic subtype [51]. IBM allows us to focus on deaths specifically linked to incident metastatic prostate cancer, which has an average survival of between 2 and 3 years [44], while reducing the potentially confounding effect of indolent prostate cancer that could have been diagnosed decades before the natural disaster [52].

### 2.4 Exposure

We used FEMA's Disaster Declarations Summaries database to identify counties that experienced a declared emergency from 2010 to 2020. We focused on disaster types with a plausible association with climate change, while non‐climate‐related natural disasters (e.g., earthquakes) were not included (Table S1). We identified a subset of these as “major disasters” as those meeting both conditions in the dataset: being tagged by FEMA as a major disaster and having individual assistance programs, as both criteria indicate severe damage to residences.

To reduce potential confounding of potential changes, natural disasters over multiple years, we focused on counties with one climate‐related natural disaster within a specific calendar year from 2012 to 2018. The year of the natural disaster was set as the index date (t = 0), and 4 years of SEER cancer data were obtained during the pre‐disaster period (2 years prior to the index date) and post‐disaster period (2 years after the index date). We then assigned categorical predictor variables t−1 and t−2 for 1 and 2 years before the natural disaster and t+1 and t+2 for 1 and 2 years after the natural disaster. Although longer survival times are seen in some clinical trials, real‐world data for metastatic disease survival for prostate cancer shows a median survival of about 24–26 months in this era [53]. Two years was therefore chosen to evaluate possible changes in mortality associated with the natural disaster.

### 2.5 Covariates

Because privacy rules censor patient level clinical and demographic information in SEER when analyzing small geographic regions like counties (e.g., age, race, tumor size, time to treatment, etc.), we were not able to include detailed demographic and treatment‐related information. For demographics, we utilized aggregate 5‐year county level data from the American Community Survey corresponding to the year of the disaster (using annual releases from 2010 to 2020).

We selected multiple demographic factors as covariates in our analysis, such as age at diagnosis, race/ethnicity, socioeconomic status, education level, marital status, as well as year of diagnosis. Although time‐invariant confounding is controlled for by design, these factors were considered to account for potential confounding from changes in demographics in the affected counties during the study period. Although case level data were not available, in clustered data such as ours, including covariates at the level of the cluster (county) for ecological studies may yield similar results to analyses controlling for individual‐level covariates [54].

### 2.6 Statistical Analysis

First, to assess for demographic shifts which may occur after natural disasters, we compared characteristics of pre‐ and post‐disaster populations using frequencies for categorical variables and means for continuous variables. We used a Wilcoxon test to compare these characteristics over time, accounting for clustering due to county [55]. A log‐linear regression model was then fit to assess the county‐level age‐adjusted incidence‐based mortality rate for advanced prostate cancer associated with years before or after the index date, adjusting for the county‐level covariates. The parameters of the log‐linear regression model were calculated using optimal weighted least squares with a log‐link, using a robust standard error to account for the repeated measures (yearly data) on the county [56]. The exponential of the regression coefficients from the log‐linear regression model are rate ratios. The ‘weights’ in the weighted least squares are the inverse of the variance of the yearly county‐level incidence‐based mortality rate; the variance is the square of the standard error of the age‐adjusted incidence‐based mortality rate that are found in the SEER database [57, 58].

While applying a self‐controlled time‐series design controls for confounding by time‐invariant factors, time‐varying confounding due to changes in care delivery that co‐occurred with the natural disasters remains a threat to validity. We therefore performed a sensitivity analysis using a control series. Because there was no “date” of a natural disaster in counties without natural disaster, we randomly assigned an index year among the 147 SEER counties that did not experience any climate‐related natural disaster with complete covariate information. We then fit regression models specifying the same lagged dummy variables before and after the index date, and covariates described previously. We used bootstrapping with 1000 repetitions to obtain point estimates and confidence intervals.

All statistical analyses were carried out using SAS 9.4 (SAS Institute Inc., Cary, North Carolina), and all tests were two‐sided with alpha = 0.05. The study received an IRB waiver 2023P003498 from the Brigham and Women's Hospital Institutional Review Board.

## 3 Results

### 3.1 Baseline Demographics and Univariable Analysis

**FIGURE 1:**
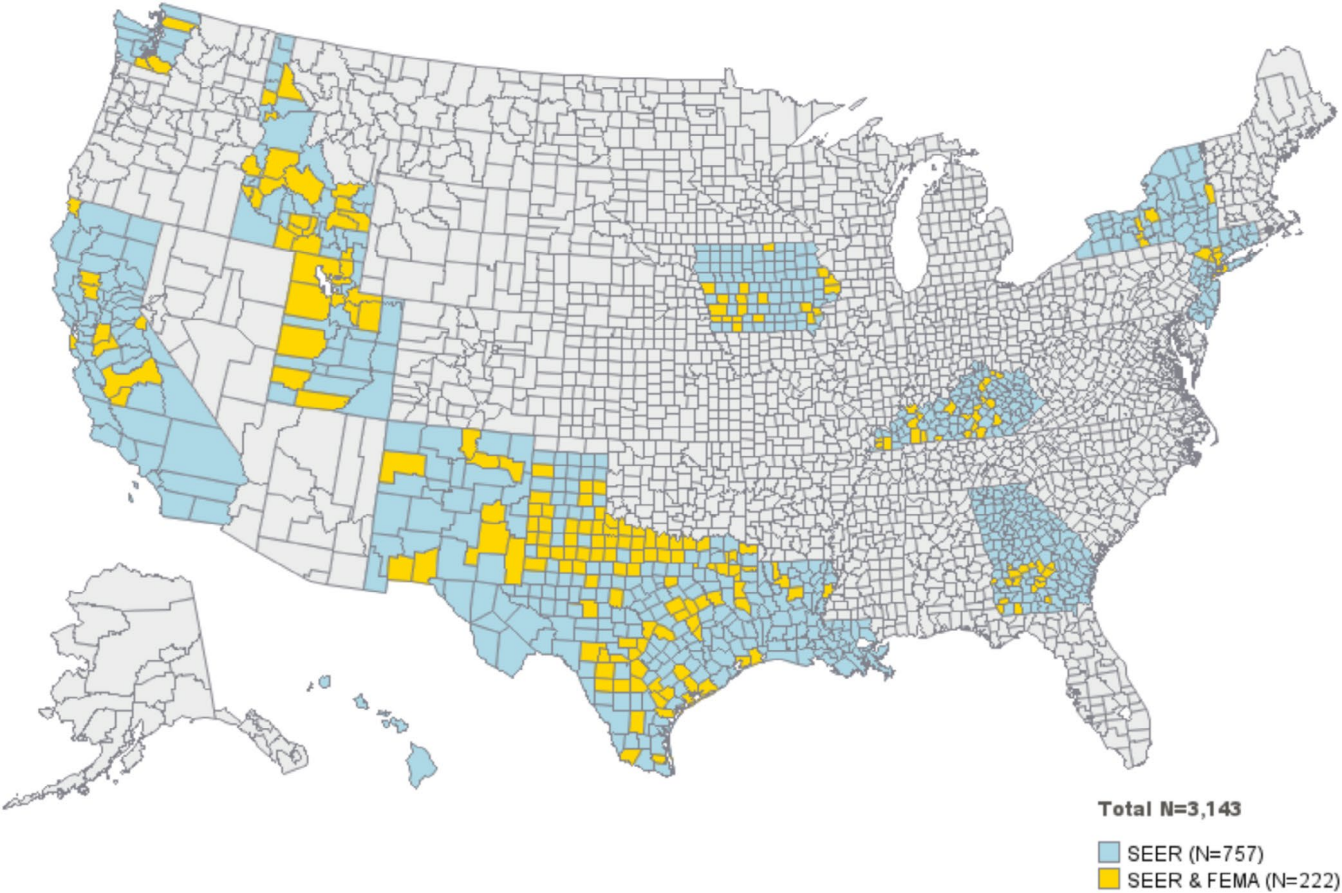
Geographic distribution of counties included in analysis.

There were 2689 counties that experienced ≥ 1 climate‐related natural disasters from 2012 to 2018; of these, 826 counties had only one climate‐related natural disaster from 2012 to 2018, and 222 counties with incidence‐based mortality data (from SEER) in the year of the natural disaster and 4 years of data pre‐ and post‐disaster. The most common declared natural disasters were fires (*n* = 17), floods (*n* = 58), hurricanes (*n* = 45) and severe storms (*n* = 97) (Table S1). The 222 counties comprising the analytic dataset were located within 11 US states, with 42% of counties reporting from Texas (Table S2). Maps of the counties retained in our analysis are presented in Figure 1. States from each of the four Census Regions (West, South, Midwest, Northeast) were included.

We included annual demographic data from the American Community Survey to assess for demographic change before, during, and after these natural disasters (Table 1). Although there were statistically significant differences in demographic characteristics between 2 years pre‐ versus post‐disaster, the absolute magnitude of these changes was small and was generally in the direction of improving sociodemographic indicators in the counties assessed. Compared to 2 years pre‐disaster, there was a decline in median county‐level percent Non‐Hispanic White population (71.1% vs. 69.0%) and increase in median percent Hispanic population (14.9% vs. 16.3%). Similarly, there was an increase in the percent with bachelor's degree or higher education (16.8% vs. 17.3%), and declines in percent uninsured < 65 years (21.6% vs. 17.0%), percent unemployed (7.1% vs. 5.7%) and percent living below the poverty line (17.0% vs. 15.5%).

**TABLE 1:** Characteristics of counties impacted by climate‐related natural disasters (counties *N* = 222).

	Two years pre‐disaster (T−2)	Baseline (T = 0)	Two years post‐disaster (T+2)	*p*
Mortality rates				
Prostate	5.5	4.5	7.0	0.082
Total population	27,240,928	27,787,120	28,343,106	
Total population over 18	20,524,298	21,033,628	21,542,534	
Male population per county Median (IQR)	10,297 (4651–30,261)	10,308 (4608–30,977)	10,388 (4677–31,225)	
Race (Pct) Median (IQR)				
Non‐Hispanic White	71.1 (54.2–86.8)	6, 69.5 (54.4–86.4)	69.0 (53.1–85.5)	0.028
Non‐Hispanic Black	2.6 (0.6–8.6)	2.7 (0.6–8.6)	2.8 (0.7–8.3)	0.62
Hispanic	14.9 (4.0–27.3)	21.2, 15.8 (4.2–28.1)	16.3 (4.4–29.0)	0.21
Non‐Hispanic Asian	0.5 (0.2–1.1)	1.4, 0.5 (0.2–1.1)	0.6 (0.2–1.3)	0.60
Non‐Hispanic Native Hawaiian & Other Pacific Islanders	0.0 (0.0–0.1)	0.0 (0.0–0.1)	0.0 (0.0–0.1)	0.77
Education (Pct) Median (IQR)				
Less than 9th Gr	6.9 (4.7–10.5)	6.4 (4.3–10.5)	5.8 (3.7–9.8)	< 0.001
Some HS education	10.1 (7.3–12.7)	9.7 (6.9–12.4)	9.2 (6.6–11.6)	< 0.001
HS graduates	32.7 (28.7–37.3)	32.9 (28.6–36.8)	32.7 (28.7–37.3)	< 0.001
Bachelor's or higher	16.8 (13.9–22.0)	16.6 (13.7–22.7)	17.3 (14.3–23.6)	< 0.001
Income and insurance coverage (Pct) Median (IQR)				
Uninsured < 65	21.6 (17.3–26.0)	20.2 (14.4–24.4)	17.0 (10.1–21.5)	< 0.001
Unemployed	7.1 (5.9–9.3)	6.6 (5.2–8.6)	5.7 (4.4–7.5)	< 0.001
Below poverty line	17.0 (12.5–21.2)	16.7 (12.2–20.9)	15.5 (12.5–20.1)	< 0.001
Receiving cash public assistance	1.9 (1.3–2.8)	1.9 (1.2–2.9)	1.8 (1.2–2.8)	0.76
Median income (In 1000 dollars) (IQR)	46.09 (38.46–50.86)	47.79 (38.82–53.81)	50.58 (42.24–56.92)	< 0.001
Housing				
Median home value, (IQR) (in 1000 dollars)	133.11 (78.30–149.40)	138.47 (80.80–157.20)	148.33 (83.2–166.80)	< 0.001

### 3.2 Multivariable Regression

The results of our linear regression model are shown in Table 2A and Table 2B. Compared to the IBM during the index year, the IBM for prostate cancer was significantly higher during the post‐disaster period. While no difference in prostate cancer IBM was observed pre‐disaster, the IBM was 15% higher (rate ratio (RR): 1.15, 95% CI: 1.02–1.30) during the first year, and 28% higher (RR: 1.28, 95% CI: 1.11–1.49) during the second‐year post‐disaster. When restricting to the 51 counties with “major” disasters, there was higher IBM during the first (RR: 1.21, 95% CI: 1.05–1.40) and second year (RR: 1.35, 95% CI: 1.17–1.55) post‐disaster compared to IBM during the index year and a larger magnitude effect compared to the full set of natural disasters.

**TABLE 2:** Association between climate‐related natural disasters and age‐adjusted mortality rates for metastatic prostate cancer.

	Counties with any climate natural disaster (*n* = 222)		Counties with major climate natural disaster^a^ (*n* = 50)	
	Rate Ratio (95% CI)	*p*	Rate ratio (95% CI)	*p*
Year				
Pre‐disaster				
T−2	1.08 (0.93–1.26)	0.31	1.14 (0.97–1.34)	0.11
T−1	1.02 (0.88–1.18)	0.79	1.01 (0.84–1.20)	0.95
Index year (T = 0)	*Ref*		*Ref*	
Post‐disaster				
T+1	1.15 (1.02–1.30)	**0.026**	1.21 (1.05–1.40)	**0.009**
T+2	1.28 (1.11–1.49)	**< 0.001**	1.35 (1.17–1.55)	**< 0.001**
Race^b^				
Pct Non‐Hispanic White	0.90 (0.83–0.97)	**< 0.009**	0.38 (0.11–1.38)	0.14
Pct Non‐Hispanic Black	0.96 (0.89–1.04)	0.32	0.45 (0.13–1.56)	0.21
Pct Hispanic	0.92 (0.86–1.00)	**0.037**	0.40 (0.11–1.45)	0.16
Pct Non‐Hispanic Asian	0.91 (0.77–1.07)	0.26	0.34 (0.08–1.37)	0.13
Education^b^				
Pct some HS education	0.85 (0.52–1.37)	0.51	1.62 (1.05–2.50)	**0.031**
Pct HS graduates	0.84 (0.63–1.12)	0.23	0.95 (0.63–1.44)	0.82
Pct bachelors or higher	1.00 (0.84–1.20)	0.99	1.18 (0.91–1.53)	0.21
Income and insurance coverage^b^				
Pct unemployed	0.93 (0.60–1.44)	0.74	1.23 (0.77–1.97)	0.39
Pct below poverty line	0.97 (0.71–1.32)	0.85	0.96 (0.62–1.50)	0.87
Pct receiving cash public assistance	1.69 (0.84–3.39)	0.14	1.34 (0.72–2.48)	0.35
Median income	1.00 (0.98–1.01)	0.34	1.00 (0.98–1.02)	0.99

^a^ Defined based on individual assistance provided. Significance for bolded text is based on *p*‐values < 0.05.

^b^ Rate ratios per 10% increase in each characteristic except median income which increases per $1000 USD annual income.

Results from our sensitivity analysis in control counties are presented in Table S3. No statistically significant associations of time since or prior to the randomly assigned index year with mortality rates for metastatic prostate cancer were observed. This suggests that time‐varying confounding is unlikely to fully explain these results.

## 4 Discussion

In this retrospective study using data from the SEER and FEMA databases, we assessed trends for adjusted prostate cancer IBM from metastatic prostate cancer in 222 counties that experienced a climate‐related natural disaster from 2010 to 2020. County‐level prostate cancer IBM was significantly higher post‐disaster compared to pre‐disaster. Furthermore, a larger increase in prostate cancer IBM was reported in the subset of counties with more severe “major” natural disasters.

This is among the first studies to report changes in prostate cancer mortality following declared climate‐related natural disasters using a broadly representative national dataset. While a large body of research exists on the downstream effects of tropical storms and hurricanes [23, 59, 60, 61], much of the existing literature focuses on infectious diseases and traumatic injury. Others have quantified the impacts of wildfires on those individuals who use durable medical equipment (DME) and other vulnerable groups [62]. Bell et al. showed higher mortality among adults with dementia after hurricanes [63], and growing evidence has accumulated regarding potential impacts of hurricanes on cardiovascular morbidity and mortality [37]. Cancer care delivery requires frequent contact with the health system and highly specialized, resource‐intensive care. Existing research suggests that variability in access and quality is a mediator of cancer outcomes [48, 64, 65], and both may be plausibly impacted by natural disasters. A recent US hospital‐based study reported worse survival for lung cancer patients who begin treatment during hurricanes [43]. A recent scoping review identified 10 studies that assessed cancer outcomes after climate‐related natural disasters; however, most of these (8/10) assessed specific hurricanes (e.g., Katrina) and the other two were narrative reviews [66].

Our work builds on this knowledge using a population‐based cancer registry linked to a high‐quality natural disaster registry. By restricting to only counties that experienced a natural disaster, with a “disaster free” wash in period, we reduce impacts of confounding by sociodemographic characteristics that may vary across counties that experienced versus did not experience a natural disaster. We further mitigated influence of time‐varying confounding by using repeated measures models and time‐varying covariates and by combining both mortality rates and impacts of advanced prostate cancer. Importantly, the finding of a “dose‐dependent” increase in mortality (greater change in mortality in counties with more severe natural disasters) provides support for the hypothesis that healthcare disruption arising from extreme weather events may impact prostate cancer mortality [67].

### 4.1 Limitations

Our data precluded linkage of cancer diagnosis and mortality dates to exact dates of natural disasters, which may introduce misclassification bias when comparing follow‐up pre‐ versus post‐disaster. We also restricted our analysis to counties that experienced a single natural disaster within our study period, and so excluded the most heavily impacted US counties (e.g., counties that experience hurricanes every 1–2 years). Both design choices would be expected to attenuate associations to the null because the greatest impact on prostate cancer IBM would be expected immediately following the disaster, and between counties with more frequent natural disasters. However, restricting the study to counties which have disaster free wash in and wash out periods with a single disaster in the index year may limit generalizability because we do not include counties that have multiple disasters in sequential years. Our study (because of our use of SEER) does not include non‐SEER reporting counties and we only included SEER participating counties shown in Figure 1.

In addition, we focused mostly on medium‐term impacts. The more long‐term impacts of natural disasters are more difficult to study with this type of study design but are likely to impact urologic diseases through several pathways [19]. For example, climate change is linked to increased air pollution due to changes in atmospheric mixing, as well as fossil fuel combustion. Both air and water pollution have been linked to higher prostate cancer incidence and mortality in many epidemiologic studies. Wildfires have been linked to prostate cancer outcomes. In Brazil, wildfire‐associated fine particulate matter (PM_2.5_) concentrations have been shown to have a significant association with prostate cancer mortality: relative risk 1.03 (95% CI 1.01–1.06) per 1‐μg/m^3^ increase [68].

Because case‐level clinical information is not available at the county level in SEER, we could not include case‐level data on specific aspects of cancer care that may be affected by hurricanes (e.g., tumor characteristics, changes in time from diagnosis to treatment, whether patients received radiation for low volume metastatic cancer). Similarly, our database did not include changes in screening patterns, health systems resources, hospital closures, or other access‐related measures that may have changed over time and are associated with prostate cancer mortality [69]. This could include mediators (e.g., closure of small hospitals due to flooding) as well as confounders (e.g., closures of small hospitals due to corporate mergers of health systems). We reported changes in demographic characteristics of the counties in our analysis post‐disaster, including a small decrease in the percentage with no HS degree and small increase in the percentage with a Bachelor's degree or higher. However, if more vulnerable and lower SES individuals are more likely to be displaced by natural disasters, we would expect this trend to generally bias our results toward the null finding as those remaining may be of slightly higher socioeconomic status (a group with generally more favorable access to cancer care and cancer outcomes) [70].

## 5 Conclusion

In this multi‐state, population‐based study, climate‐related natural disasters were associated with higher prostate cancer mortality rates in the 2‐year post‐disaster period. Associations were larger in magnitude for more severe disasters. This pattern was not seen in a set of control counties without natural disasters. These results suggest that greater frequency of extreme weather events could be associated with higher prostate cancer mortality, and a need to identify interventions within the healthcare system to reduce care disruption for this large group of cancer patients. Our findings highlight the critical, national need for a climate resilient cancer care system.

## Author Contributions

**Alexander P. Cole:** conceptualization (equal), formal analysis (equal), writing – original draft (equal). **Zhiyu Qian:** formal analysis (equal), writing – review and editing (equal). **Yu‐Jen Chen:** formal analysis (equal), investigation (equal). **Edoardo Beatrici:** data curation (equal), formal analysis (equal). **Rohit Acharya:** data curation (equal), writing – review and editing (equal). **Danesha Daniels:** writing – review and editing (equal). **Prokar Dasgupta:** writing – review and editing (equal). **Adam S. Kibel:** writing – review and editing (equal). **Stuart R. Lipsitz:** writing – review and editing (equal). **Quoc‐Dien Trinh:** resources (equal), supervision (equal), writing – review and editing (equal). **Hari S. Iyer:** writing – original draft (equal).

## Funding

APC reports research funding from the Bruce A Beal and Robert L Beal surgical fellowship of the BWH Department of Surgery, from the Prostate Cancer Foundation and American Cancer Society (#23YOUN25) and from a Physician Research Award from the Department of Defense Congressionally Directed Medical Research Program (#PC220342).

## Conflicts of Interest

Q.‐D.T. reports personal fees from Astellas, Bayer, Intuitive Surgical, Janssen, Novartis, and Pfizer outside the submitted work. Q.‐D.T. reports research funding from the American Cancer Society, the Defense Health Agency and Pfizer Global Medical Grants. A.P.C. reports research funding from the American Cancer Society and Pfizer Global Medical Grants and proctoring fees from EDAP/Focal One. P.D. reports funding from Proximie and MysteryVibe. The funders were not involved in the writing of this manuscript.

## Supporting information


**Table S1:** Counts of natural disasters and disaster types from Federal Emergency Management Assistance Database.
**Table S2:** US States included in analytic cohort (*n* = 222 declared natural disasters).
**Table S3:** Association between climate‐related natural disasters and age‐adjusted mortality rates for metastatic prostate cancer comparing 222 counties with a disaster (“Disaster”) to 147 counties without a disaster (“Control”).

## Data Availability

The data that support the findings of this study are derived from the Surveillance, Epidemiology, and End Results (SEER) program of the National Cancer Institute. This study uses the Federal Emergency Management Agency's Disaster Declarations Summaries but is not endorsed by FEMA. The Federal Government or FEMA cannot vouch for the data or analyses derived from these data after the data have been retrieved from the Agency's website(s).

## References

[1] P. R. Epstein , “Climate Change and Human Health,” New England Journal of Medicine 353 (2005): 1433–1436. 16207843 10.1056/NEJMp058079

[2] World Health Organization , “Climate Change and Health: Report of the Secretariat,” 2008.

[3] L. M. Nogueira , T. E. Crane , A. P. Ortiz , H. D'Angelo , and G. Neta , “Climate Change and Cancer,” Cancer Epidemiology, Biomarkers & Prevention 32 (2023): 869–875. 10.1158/1055-9965.EPI-22-1234 37184574

[4] E. Bernicker , S. D. Averbuch , S. Edge , et al., “Climate Change and Cancer Care: A Policy Statement From ASCO,” JCO Oncology Practice 20, no. 2 (2023): 178–186. 38011607 10.1200/OP.23.00637

[5] A. P., Cole , Z. Qian , N. Gupta , et al., “Urology on a changing planet: links between climate change and urological disease,” Nature Reviews Urology 22, no. 4 (2025): 208–222, 10.1038/s41585-024-00979-4. 39875561

[6] R. L. Siegel , K. D. Miller , N. S. Wagle , and A. Jemal , “Cancer Statistics, 2023,” CA: A Cancer Journal for Clinicians 73 (2023): 17–48. 36633525 10.3322/caac.21763

[7] R. T. Dess , H. E. Hartman , B. A. Mahal , et al., “Association of Black Race With Prostate Cancer‐Specific and Other‐Cause Mortality,” JAMA Oncology 5 (2019): 975–983. 31120534 10.1001/jamaoncol.2019.0826 PMC6547116

[8] A. P. Cole , N. Gupta , and S. Loeb , The Plant‐Based Prescription: How Dietary Change Can Improve Both Urological and Planetary Health (Elsevier, 2023). 10.1016/j.eururo.2023.06.020 37451898

[9] M. S. Leapman , C. L. Thiel , I. O. Gordon , et al., “Environmental Impact of Prostate Magnetic Resonance Imaging and Transrectal Ultrasound Guided Prostate Biopsy,” European Urology 83 (2023): 463–471. 36635108 10.1016/j.eururo.2022.12.008

[10] A. P. Cole and S. Loeb , “Dietary and Lifestyle Recommendations That Align Patient and Planetary Health,” European Urology Focus 9 (2023): 869–872. 37770372 10.1016/j.euf.2023.09.007

[11] J. S. Jagai , L. C. Messer , K. M. Rappazzo , C. L. Gray , S. C. Grabich , and D. T. Lobdell , “County‐Level Cumulative Environmental Quality Associated With Cancer Incidence,” Cancer 123 (2017): 2901–2908. 28480506 10.1002/cncr.30709 PMC6121813

[12] L. M. K. Youogo , M. E. Parent , P. Hystad , et al., “Ambient Air Pollution and Prostate Cancer Risk in a Population‐Based Canadian Case‐Control Study,” Environmental Epidemiology 6 (2022): e219. 35975163 10.1097/EE9.0000000000000219 PMC9374191

[13] H. S. Iyer , P. James , L. Valeri , et al., “The Association Between Neighborhood Greenness and Incidence of Lethal Prostate Cancer: A Prospective Cohort Study,” Environmental Epidemiology 4 (2020): e091. 32656487 10.1097/EE9.0000000000000091 PMC7319229

[14] H. S. Iyer , K. H. Kensler , J. B. Vaselkiv , et al., “Associations Between Etiologic or Prognostic Tumor Tissue Markers and Neighborhood Contextual Factors in Male Health Professionals Diagnosed With Prostate Cancer,” Cancer Epidemiology, Biomarkers & Prevention:OF1‐OF4 32, no. 8 (2023): 1120–1123. 10.1158/1055-9965.EPI-23-0217 PMC10527012 37249585

[15] H. S. Iyer , J. B. Vaselkiv , K. H. Stopsack , et al., “Influence of Neighborhood Social and Natural Environment on Prostate Tumor Histology in a Cohort of Male Health Professionals,” American Journal of Epidemiology 192, no. 9 (2023): 1485–1498. 37139568 10.1093/aje/kwad112 PMC10948945

[16] H. T. Vigneswaran , J. S. Jagai , D. T. Greenwald , et al., “Association Between Environmental Quality and Prostate Cancer Stage at Diagnosis,” Prostate Cancer and Prostatic Diseases 24 (2021): 1129–1136. 33947975 10.1038/s41391-021-00370-z

[17] L. Multigner , J. R. Ndong , A. Giusti , et al., “Chlordecone Exposure and Risk of Prostate Cancer,” Journal of Clinical Oncology 28 (2010): 3457–3462. 20566993 10.1200/JCO.2009.27.2153

[18] M. J. Zare Sakhvidi , J. Yang , J. Siemiatycki , et al., “Greenspace Exposure and Cancer Incidence: A 27‐Year Follow‐Up of the French GAZEL Cohort,” Sci Total Environ 787 (2021): 147553. 33989869 10.1016/j.scitotenv.2021.147553

[19] A. P. Cole , Z. Qian , N. Gupta , et al., “Urology on a Changing Planet: Links Between Climate Change and Urological Disease,” Nature Reviews. Urology 22 (2025): 208–222. 39875561 10.1038/s41585-024-00979-4

[20] M. Friedrich , “Determining Health Effects of Hazardous Materials Released During Hurricane Harvey,” JAMA 318 (2017): 2283–2285. 29188269 10.1001/jama.2017.15558

[21] J. Ponting , T. J. Kelly , A. Verhoef , M. J. Watts , and T. Sizmur , “The Impact of Increased Flooding Occurrence on the Mobility of Potentially Toxic Elements in Floodplain Soil–A Review,” Science of the Total Environment 754 (2021): 142040. 32916489 10.1016/j.scitotenv.2020.142040

[22] S. Tuminello , W. Lieberman‐Cribbin , S. Kerath , et al., “Exposure to Chemical and Toxic Elements Following Hurricane Harvey,” Environmental Epidemiology 3 (2019): 239–240.

[23] T. B. Erickson , J. Brooks , E. J. Nilles , P. N. Pham , and P. Vinck , “Environmental Health Effects Attributed to Toxic and Infectious Agents Following Hurricanes, Cyclones, Flash Floods and Major Hydrometeorological Events,” Journal of Toxicology and Environmental Health, Part B 22 (2019): 157–171. 10.1080/10937404.2019.1654422 31437111

[24] K. Steenland and A. Winquist , “PFAS and Cancer, a Scoping Review of the Epidemiologic Evidence,” Environmental Research 194 (2021): 110690. 33385391 10.1016/j.envres.2020.110690 PMC7946751

[25] M. Di Donato , G. Cernera , P. Giovannelli , et al., “Recent Advances on Bisphenol‐A and Endocrine Disruptor Effects on Human Prostate Cancer,” Molecular and Cellular Endocrinology 457 (2017): 35–42. 28257827 10.1016/j.mce.2017.02.045

[26] W.‐Y. Hu , G.‐B. Shi , D.‐P. Hu , J. L. Nelles , and G. S. Prins , “Actions of Estrogens and Endocrine Disrupting Chemicals on Human Prostate Stem/Progenitor Cells and Prostate Cancer Risk,” Molecular and Cellular Endocrinology 354 (2012): 63–73. 21914459 10.1016/j.mce.2011.08.032 PMC3249013

[27] A. Lacouture , C. Lafront , C. Peillex , M. Pelletier , and É. Audet‐Walsh , “Impacts of Endocrine‐Disrupting Chemicals on Prostate Function and Cancer,” Environmental Research 204 (2022): 112085. 34562481 10.1016/j.envres.2021.112085

[28] G. S. Prins , S. H. Ye , L. Birch , et al., “Prostate Cancer Risk and DNA Methylation Signatures in Aging Rats Following Developmental BPA Exposure: A Dose‐Response Analysis,” Environmental Health Perspectives 125 (2017): 077007. 28728135 10.1289/EHP1050 PMC5744650

[29] S. Koutros , L. E. Beane Freeman , J. H. Lubin , et al., “Risk of Total and Aggressive Prostate Cancer and Pesticide Use in the Agricultural Health Study,” American Journal of Epidemiology 177 (2013): 59–74. 23171882 10.1093/aje/kws225 PMC3590039

[30] H. S. Iyer , J. E. Hart , P. James , et al., “Impact of Neighborhood Socioeconomic Status, Income Segregation, and Greenness on Blood Biomarkers of Inflammation,” Environment International 162 (2022): 107164. 35255255 10.1016/j.envint.2022.107164 PMC8985077

[31] A. I. Egorov , S. M. Griffin , J. N. Styles , et al., “Time Outdoors and Residential Greenness Are Associated With Reduced Systemic Inflammation and Allostatic Load,” Environmental Pollution 344 (2024): 123408. 38278402 10.1016/j.envpol.2024.123408

[32] A. I. Egorov , S. M. Griffin , R. R. Converse , et al., “Greater Tree Cover Near Residence Is Associated With Reduced Allostatic Load in Residents of Central North Carolina,” Environmental Research 186 (2020): 109435. 32315826 10.1016/j.envres.2020.109435 PMC7584403

[33] I. Kayyal‐Tarabeia , Y. Michael , I. M. Lensky , M. Blank , and K. Agay‐Shay , “Residential Greenness and Site‐Specific Cancer: A Registry Based Cohort of 144,427 Participants With a 21‐Years of Follow‐Up, Tel‐Aviv District, Israel,” Environmental Research 212 (2022): 113460. 35561833 10.1016/j.envres.2022.113460

[34] Y.‐J. Huang , P.‐H. Lee , L.‐C. Chen , B. C. Lin , C. Lin , and T. C. Chan , “Relationships Among Green Space, Ambient Fine Particulate Matter, and Cancer Incidence in Taiwan: A 16‐Year Retrospective Cohort Study,” Environmental Research 212 (2022): 113416. 35523280 10.1016/j.envres.2022.113416

[35] Y. J. Lee , W. Q. Loh , T. K. Dang , et al., “Determinants of Residential Greenness and Its Association With Prostate Cancer Risk: A Case‐Control Study in Singapore,” Environmental Research 237 (2023): 116903. 37598842 10.1016/j.envres.2023.116903

[36] K. L. Ebi , J. Vanos , J. W. Baldwin , et al., “Extreme Weather and Climate Change: Population Health and Health System Implications,” Annual Review of Public Health 42 (2021): 293–315. 10.1146/annurev-publhealth-012420-105026 PMC9013542 33406378

[37] A. K. Ghosh , M. R. Demetres , B. P. Geisler , et al., “Impact of Hurricanes and Associated Extreme Weather Events on Cardiovascular Health: A Scoping Review,” Environmental Health Perspectives 130 (2022): 116003. 36448792 10.1289/EHP11252 PMC9710380

[38] P. V. Barbosa , I. C. Thomas , S. Srinivas , et al., “Overall Survival in Patients With Localized Prostate Cancer in the US Veterans Health Administration: Is PIVOT Generalizable?,” European Urology 70 (2016): 227–230. 26948397 10.1016/j.eururo.2016.02.037 PMC4927398

[39] C. A. Sacks , A. S. Kesselheim , and M. Fralick , “The Shortage of Normal Saline in the Wake of Hurricane Maria,” JAMA Internal Medicine 178 (2018): 885–886. 29799949 10.1001/jamainternmed.2018.1936

[40] O. R. de Arzola , “Emergency Preparedness and Hurricane Maria: The Experience of a Regional Academic Medical Center in Southwest Puerto Rico,” Journal of Graduate Medical Education 10 (2018): 477–480. 30154988 10.4300/JGME-D-18-00547.1 PMC6108355

[41] A. J. Hertelendy and G. R. Ciottone , “Averting Flood‐Related Deaths and Injuries From Hurricanes: Enhancing Hospital Resilience,” Lancet Regional Health—Americas 40 (2024): 100930. 39507521 10.1016/j.lana.2024.100930 PMC11539662

[42] D. Aguero and D. Allen , “Weathering the Storm: Commentary on the Hurricane Helene IV Fluid Shortage,” Journal of Pediatric Pharmacology and Therapeutics 29 (2024): 667–669. 10.5863/1551-6776-29.6.667 PMC11627566 39659851

[43] L. M. Nogueira , L. Sahar , J. A. Efstathiou , A. Jemal , and K. R. Yabroff , “Association Between Declared Hurricane Disasters and Survival of Patients With Lung Cancer Undergoing Radiation Treatment,” JAMA 322 (2019): 269–271. 31310288 10.1001/jama.2019.7657 PMC6635902

[44] C. Corsini , H. Garmo , A. W. Orrason , R. Gedeborg , P. Stattin , and M. Westerberg , “Survival Trend in Individuals With de Novo Metastatic Prostate Cancer After the Introduction of Doublet Therapy,” JAMA Network Open 6 (2023): e2336604. 37782498 10.1001/jamanetworkopen.2023.36604 PMC10546238

[45] J. D. Sammon , E. C. Serrell , P. Karabon , et al., “Prostate Cancer Screening in Early Medicaid Expansion States,” Journal of Urology 199 (2018): 81–88. 28765069 10.1016/j.juro.2017.07.083

[46] K. Alkhatib , M. Labban , L. Briggs , et al., “Does Veteran Status Mitigate Racial Disparities in Prostate Cancer Screening? Analysis of Prostate Specific Antigen Screening Patterns in the 2018 Behavioral Risk Factor Surveillance System Data,” Journal of Urology 207 (2022): 993–1000. 34967663 10.1097/JU.0000000000002379

[47] A. P. Cole , C. Lu , M. J. Krimphove , et al., “Comparing the Association Between Insurance and Mortality in Ovarian, Pancreatic, Lung, Colorectal, Prostate, and Breast Cancers,” Journal of the National Comprehensive Cancer Network 17 (2019): 1049–1058. 31487683 10.6004/jnccn.2019.7296

[48] M. J. Krimphove , S. A. Fletcher , A. P. Cole , et al., “Quality of Care in the Treatment of Localized Intermediate and High Risk Prostate Cancer at Minority Serving Hospitals,” Journal of Urology 27 (2018): 4300. 10.1016/j.juro.2018.10.024 30414956

[49] FEMA , “FEMA: OpenFEMA Dataset: Disaster Declarations Summaries—v2,” 2023.

[50] J. L. Bernal , S. Cummins , and A. Gasparrini , “Interrupted Time Series Regression for the Evaluation of Public Health Interventions: A Tutorial,” International Journal of Epidemiology 46 (2017): 348–355. 27283160 10.1093/ije/dyw098 PMC5407170

[51] K. C. Chu , B. A. Miller , E. J. Feuer , and B. F. Hankey , “A Method for Partitioning Cancer Mortality Trends by Factors Associated With Diagnosis: An Application to Female Breast Cancer,” Journal of Clinical Epidemiology 47 (1994): 1451–1461. 7730854 10.1016/0895-4356(94)90089-2

[52] E. J. Feuer , R. M. Merrill , and B. F. Hankey , “Cancer Surveillance Series: Interpreting Trends in Prostate Cancer—Part II: Cause of Death Misclassification and the Recent Rise and Fall in Prostate Cancer Mortality,” Journal of the National Cancer Institute 91 (1999): 1025–1032. 10379965 10.1093/jnci/91.12.1025

[53] J. T. Helgstrand , M. A. Røder , N. Klemann , et al., “Trends in Incidence and 5‐Year Mortality in Men With Newly Diagnosed, Metastatic Prostate Cancer—A Population‐Based Analysis of 2 National Cohorts,” Cancer 124 (2018): 2931–2938. 29723398 10.1002/cncr.31384

[54] H. R. Guo , S. R. Lipsitz , H. Hu , and R. R. Monson , “Using Ecological Data to Estimate a Regression Model for Individual Data: The Association Between Arsenic in Drinking Water and Incidence of Skin Cancer,” Environmental Research 79 (1998): 82–93. 9841806 10.1006/enrs.1998.3863

[55] S. Natarajan , S. R. Lipsitz , G. M. Fitzmaurice , et al., “An Extension of the Wilcoxon Rank Sum Test for Complex Sample Survey Data,” Journal of the Royal Statistical Society: Series C: Applied Statistics 61 (2012): 653–664. 23913985 10.1111/j.1467-9876.2011.01028.x PMC3729471

[56] H. White , “Maximum Likelihood Estimation of Misspecified Models,” Econometrica 50 (1982): 1–25.

[57] D. A. Bloch and L. E. Moses , “Nonoptimally Weighted Least Squares,” American Statistician 42 (1988): 50–53.

[58] S. R. Lipsitz , “Methods for Estimating the Parameters of a Linear Model for Ordered Categorical Data,” Biometrics 48 (1992): 271–281. 1581487

[59] D. D. Saulnier , K. B. Ribacke , and J. von Schreeb , “No Calm After the Storm: A Systematic Review of Human Health Following Flood and Storm Disasters,” Prehospital and Disaster Medicine 32 (2017): 568–579. 28606191 10.1017/S1049023X17006574

[60] K. Alderman , L. R. Turner , and S. Tong , “Floods and Human Health: A Systematic Review,” Environment International 47 (2012): 37–47. 22750033 10.1016/j.envint.2012.06.003

[61] R. M. Parks , J. Benavides , G. B. Anderson , et al., “Association of Tropical Cyclones With County‐Level Mortality in the US,” JAMA 327 (2022): 946–955. 35258534 10.1001/jama.2022.1682 PMC8905400

[62] H. McBrien , S. T. Rowland , T. Benmarhnia , S. Y. Tartof , B. Steiger , and J. A. Casey , “Wildfire Exposure and Health Care Use Among People Who Use Durable Medical Equipment in Southern California,” Epidemiology 34 (2023): 700–711. 37255240 10.1097/EDE.0000000000001634 PMC10524711

[63] S. A. Bell , M. L. Miranda , J. P. Bynum , J. P. W. Bynum , and M. A. Davis , “Mortality After Exposure to a Hurricane Among Older Adults Living With Dementia,” JAMA Network Open 6 (2023): e232043. 36881412 10.1001/jamanetworkopen.2023.2043 PMC9993175

[64] A. P. Cole , D. D. Nguyen , A. Meirkhanov , et al., “Association of Care at Minority‐Serving vs Non‐Minority‐Serving Hospitals With Use of Palliative Care Among Racial/Ethnic Minorities With Metastatic Cancer in the United States,” JAMA Network Open 2 (2019): e187633. 30707230 10.1001/jamanetworkopen.2018.7633 PMC6484582

[65] D. D. Nguyen , M. Paciotti , M. Marchese , et al., “Effect of Medicaid Expansion on Receipt of Definitive Treatment and Time to Treatment Initiation by Racial and Ethnic Minorities and at Minority‐Serving Hospitals: A Patient‐Level and Facility‐Level Analysis of Breast, Colon, Lung, and Prostate Cancer,” JCO Oncology Practice 17 (2021): e654–e665. 33974827 10.1200/OP.21.00010

[66] K. A. Lynch and A. A. Merdjanoff , “Impact of Disasters on Older Adult Cancer Outcomes: A Scoping Review,” JCO Global Oncology 9 (2023): e2200374. 37290025 10.1200/GO.22.00374 PMC10497294

[67] A. B. Hill , The Environment and Disease: Association or Causation? (Sage Publications, 1965).

[68] P. Yu , R. Xu , S. Li , et al., “Exposure to Wildfire‐Related PM2.5 and Site‐Specific Cancer Mortality in Brazil From 2010 to 2016: A Retrospective Study,” PLoS Medicine 19 (2022): e1004103. 36121854 10.1371/journal.pmed.1004103 PMC9529133

[69] B. V. Stone , M. Labban , E. Beatrici , et al., “The Association of County‐Level Prostate‐Specific Antigen Screening With Metastatic Prostate Cancer and Prostate Cancer Mortality,” European Urology Oncology 7, no. 3 (2023): 563–569. 38155059 10.1016/j.euo.2023.11.020

[70] A. P. Cole , P. Herzog , H. S. Iyer , et al., “Racial Differences in the Treatment and Outcomes for Prostate Cancer in Massachusetts,” Cancer 127 (2021): 2714–2723. 33999405 10.1002/cncr.33564 PMC9107927

