# Supplementary material for: Evaluating Potential Impacts of Climate‐Related Natural Disasters on Subsequent Prostate Cancer Mortality
Source: Cancer Med. 2026 Feb 11;15(2):e71618. doi: 10.1002/cam4.71618 (PMC12895080; doi:10.1002/cam4.71618)
Supplement: Supplementary file 1 — Table S1: Counts of natural disasters and disaster types from Federal Emergency Management Assistance Database. Table S2: US States included in analytic cohort (n = 222 declared natural disasters). Table S3: Association between climate‐related natural disasters and age‐adjusted mortality rates for metastatic prostate cancer comparing 222 counties with a disaster (“Disaster”) to 147 counties without a disaster (“Control”). [file CAM4-15-e71618-s001.docx]

**Supplemental Table 1. Counts of Natural Disasters and Disaster Types from Federal Emergency Management Assistance Database**

|  | **(A) Counties with ≥ 1 Natural Disaster (2012-2018)** | | **(B) Counties with = 1 Natural Disaster (2012-2018*)** | | **(C) Counties with = 1 Natural Disaster (2012-2018* and Cancer Mortality Rates in SEER)** | |
| --- | --- | --- | --- | --- | --- | --- |
| **Type of Natural Disaster** | **N** | **%** | **N** | **%** | **N** | **%** |
| Coastal Storm | 12 | 0% | 0 | 0% | 0 | 0% |
| Drought | 0 | 0% | 0 | 0% | 0 | 0% |
| Fire | 224 | 5% | 38 | 5% | 17 | 8% |
| Flood | 941 | 21% | 214 | 26% | 58 | 26% |
| Freezing | 1 | 0% | 0 | 0% | 0 | 0% |
| Hurricane | 1,104 | 24% | 137 | 17% | 45 | 20% |
| Mud/Landslide | 25 | 1% | 0 | 0% | 0 | 0% |
| Severe Ice Storm | 278 | 6% | 30 | 4% | 0 | 0% |
| Severe Storm | 1,601 | 35% | 363 | 44% | 97 | 44% |
| Snowstorm | 244 | 5% | 34 | 4% | 5 | 2% |
| Tornado | 86 | 2% | 10 | 1% | 0 | 0% |
| Tropical Storm | 0 | 0% | 0 | 0% | 0 | 0% |
| Typhoon | 4 | 0% | 0 | 0% | 0 | 0% |
| Winter Storm | 0 | 0% | 0 | 0% | 0 | 0% |
| **Number of Declared Disasters** | **4,520** | **100%** | **826** | **100%** | **222** | **100%** |
| Number of Counties N | 2,689 |  | 826 |  | 222 |  |

*And no natural disasters in 2010-2011 “wash-in” and 2019-2020 “wash out” periods

**Supplemental Table 2. US States included in Analytic Cohort (n=222 declared natural disasters)**

| **State** | **County N** | **Percent** |
| --- | --- | --- |
| Texas | 93 | 42% |
| Kentucky | 23 | 10% |
| Iowa | 19 | 9% |
| Idaho | 19 | 9% |
| Georgia | 17 | 8% |
| New York | 15 | 7% |
| Utah | 11 | 5% |
| California | 9 | 4% |
| New Mexico | 9 | 4% |
| Louisiana | 4 | 2% |
| Washington | 3 | 1% |
| **Total** | **222** | **100%** |

**Supplemental Table 3. Association Between Climate Related Natural Disasters and Age Adjusted Mortality Rates for Metastatic Prostate Cancer Comparing 222 Counties with a Disaster (“Disaster”) to 147 Counties without a Disaster (“Control”)**

|  | **Rate Ratio (95% CI)** | ***P* value** |
| --- | --- | --- |
| **Year (Disaster)** |  |  |
| *T -2* | 1.07 (0.92 – 1.24) | 0.36 |
| T -1 | 1.02 (0.88 – 1.17) | 0.81 |
| Year of Natural Disaster | *Ref* |  |
| T + 1 | **1.15 (1.02 – 1.30)** | **0.028** |
| T + 2 | **1.28 (1.11 – 1.47)** | **<0.001** |
| **Year (Control)** |  |  |
| *T -2* | 0.89 (0.56 – 1.41) | 0.61 |
| T -1 | 0.97 (0.60 – 1.57) | 0.89 |
| Year of “Natural Disaster” | *Ref* |  |
| T + 1 | 1.08 (0.66 – 1.76) | 0.76 |
| T + 2 | 1.09 (0.74 – 1.60) | 0.65 |
|  |  |  |
| **Race** |  |  |
| Pct Non-Hispanic White | 0.90 (0.84 – 0.98) | 0.001 |
| Pct Non-Hispanic Black | 0.93 (0.86 – 1.01) | 0.11 |
| Pct Hispanic | 0.93 (0.87 – 0.99) | 0.035 |
| Pct Non-Hispanic Asian | 0.93 (0.81 – 1.07) | 0.31 |
|  |  |  |
| **Education** |  |  |
| Pct Some HS Education | 0.84 (0.56 – 1.28) | 0.43 |
| Pct HS Graduates | 0.93 (0.73 – 1.19) | 0.58 |
| Pct Bachelors or Higher | 1.06 (0.89 – 1.25) | 0.51 |
|  |  |  |
| **Income and Insurance Coverage** |  |  |
| Median Income | 0.99 (0.98 – 1.00) | 0.18 |
| Pct Unemployed | 0.92 (0.64 – 1.31) | 0.63 |
| Pct Below Poverty Line | 0.97 (0.74 – 1.27) | 0.82 |
| Pct Receiving Cash Public Assistance | 1.89 (0.99 – 3.62) | 0.055 |
| *Defined based on individual assistance provided  ** Rate ratios per 10% increase in each characteristic except median income which is increase per $1,000 USD annual income | | |
